# Evaluating the Potential Use of Smartphone Apps for Diabetes Self-Management in an Underserved Population: A Qualitative Approach

**DOI:** 10.3390/ijerph18189886

**Published:** 2021-09-20

**Authors:** Jenny Luo, Shelley White-Means

**Affiliations:** College of Graduate Health Sciences, University of Tennessee Health Science Center, Memphis, TN 38163, USA; swhiteme@uthsc.edu

**Keywords:** mHealth, ehealth, smartphone apps, diabetes, health disparities, self-management

## Abstract

Health disparities cause a higher rate of diabetes development in poor and minority groups and also limit the care these people receive. Smartphone applications (apps) may be a low-cost, accessible resource to patients with diabetes who experience barriers to traditional health care. Currently, little is known about using health apps to help underserved patients in the United States. This study aimed to investigate the willingness to use diabetes apps in patients with limited access to primary care providers. Fifteen personal interviews were collected and analyzed according to the interpretative phenomenological analysis framework. The interviews produced three overall themes: (1) Despite having little previous knowledge about health apps, patients were all willing to try at least one diabetes-related app; (2) app functions should be individualized to each patient’s needs for maximum benefit; and (3) barriers to app use were varied but commonly included knowledge and technological challenges and security issues. Underserved patients with diabetes expressed a willingness to try health apps, despite limited experience with the technology. Choosing apps individualized to each patient’s needs, instead of a blanket multifunctional app, would provide the greatest benefit for patient-driven diabetes management. Smartphone apps may be a feasible, low-cost resource for patients with limited access to traditional healthcare.

## 1. Introduction

Diabetes mellitus is a chronic condition with a growing prevalence and high economic burden in the United States. About one in 10 adults, or over 30 million Americans, currently live with diabetes. Left unmanaged, the condition can lead to a variety of serious and costly complications including renal failure, amputations, and blindness. In total, diabetes contributes to approximately $330 billion in annual medical and lost productivity costs [1].

Diabetes management itself is a lifelong process. Effective disease control involves more than just regular doctors’ office visits; it includes a variety of patient-driven factors ranging from diet control to being able to recognize symptoms of a hyper- or hypoglycemic episode. Not all patients with diabetes, however, have access to the resources needed for successful disease management. Health disparities often lead to a higher frequency of diabetes in poor and minority groups. Compared to whites, blacks, Hispanics, and American Indians all have higher rates of being uninsured in the United States. Low-income populations also have a higher rate of being uninsured versus those with more income [2]. Consequently, these low-income and minority populations have limited access to care and poorer-quality care. More African Americans, Hispanics, and Asians consider the emergency room as their main source of healthcare compared to whites, and, thus, were less likely than whites to visit a primary care provider or private practice [3]. Such access challenges lead to costly emergency room overutilization and poor glycemic control. Since underserved groups have limited access to traditional routes of healthcare, providers should explore other ways to reach this patient population.

Mobile health (mHealth), or the delivery of health care through mobile devices, has grown rapidly in the last decade. The mHealth presents a possible low-cost platform to offer patients care and health information. Providing health resources via mobile devices like smartphones may help address the aforementioned health disparities. Unlike traditional means of healthcare, the smartphone is an accessible resource and readily used by minority and low-income populations [4]. Over 95% of American adults own a cell phone; of these, 77% own a smartphone [5]. Additionally, more minorities (70% of blacks, 71% of Hispanics) own a smartphone compared to whites (61%). While smartphone ownership rises in conjunction with income, even half of adults in the lowest income tier, <$30,000 annually, indicated ownership. The vast majority of smartphone owners also consider their devices invaluable, with 93% stating their phones as helpful and 80% as worth the cost [4].

Smartphone software applications (“apps”) are a potential tool individuals can use for their diabetes self-management. A current search revealed over 1000 commercial apps related to diabetes available in the US Apple iTunes and Google Android Play stores. With most costing as little as 99 cents or nothing to download, smartphone apps have the potential to become a valuable, low-cost resource underserved patients with diabetes can use in their day-to-day health routines.

As the application of smartphone apps to health-related activities is a relatively new area of study, there are a limited number of randomized, controlled US studies available that have examined the effect of mobile app use to augment diabetes self-management. What does exist, however, indicates a potential benefit mobile apps may provide to patients’ diabetes self-management. One study examined the use of WellDoc, an FDA-approved smartphone app that provides patients with a medication adherence tracker, hyper- and hypoglycemia treatment recommendations, and real-time feedback from physicians on their current glucose readings. In a randomized, controlled trial, the study compared using the app to standard care via doctors’ office visits. Results showed that after 3 months, participants who used WellDoc had a signification drop in their hemoglobin A1c levels (2.03% decrease compared to 0.68% for the control group). App users also had more modifications (84%) from their physicians to their medications compared to the control group (23%) [6]. A second study looked at the use of a popular smartphone app, Glucose Buddy. The app itself is commercially available for free via both the iTunes and Google Play store and is a multifunctional tracker for blood sugar, medications, weight, blood pressure, and carbohydrates. At the study’s conclusion, participants who used the app demonstrated a decrease of 1.28% in their A1c levels compared to the control group, who showed no improvement [7].

Internationally, results from several randomized, controlled studies have indicated a positive impact on clinical outcomes and patient satisfaction with diabetes app use. For instance, researchers examining the use of the DialBetics smartphone software found a greater reduction both in A1c levels (0.4% vs. 0.1%) and fasting glucose (−5.5 mg/dL vs. +16.9 mg/dL) with DialBetics use compared to no use at all [8]. Another study compared the use of the Monica app, which provides graphs and feedback communication from nurses based on entered blood pressure, weight, exercise, and glucose values, to standard care with diabetes education and counseling. The app use group saw a significantly greater A1c decrease (−0.4% vs. +0.036%) and weight reduction (−2.1 kg vs. +0.4 kg) [9]. Other studies evaluating diabetes management behaviors have also reported promising results. One, which looked at the use of the Diabetes Under Control digital diary app in comparison to a standard paper diary, found no difference in quality-of-life measures between the two groups [10]. Another study evaluated medication titration based on real-time feedback from the t+diabetes/t+medical digital diary app against titration via standard care. Results showed that app use led to more tailoring of medication regimens; 86% of app users vs. 57% of standard care patients made changes to their medications based on glucose readings [11].

The goal of this study was to determine the interest in and feasibility of using smartphone apps to help underserved patients in the Memphis, TN, area manage their diabetes at home.

Specifically, the study aimed to meet the following objectives:

(1) Obtain the attitudes about and interest in mHealth app usage from a low-income, minority population;
(2) Assess patients’ interest in various app functions for diabetes self-management;
(3) Determine any barriers to use or concerns patients may have about using an app.

## 2. Materials and Methods

### 2.1. Rationale for Qualitative Methods

Qualitative research at its core examines how people create and value a social experience [12]. Unlike quantitative inquiry, which reduces worldly observations down to structured numeric values, qualitative research involves a more linguistic approach, which allows for more flexibility and openness with the data [13]. Exploring different patients’ perceptions of mobile health allows a fuller understanding of the factors and influences that make people open to using their smartphones for health management. Qualitative research permits the researcher to study and interpret behavior in context or a natural setting [12,14]; this means that people’s families, work, and backgrounds are equally important as the outcome of interest itself. This notion was especially relevant to this study, which sought to understand patients’ reception of mobile health technology in the setting of limited access to traditional healthcare providers.

### 2.2. Research Framework

Phenomenology served as the primary methodology for this study. This particular method of inquiry investigates the way people experience and think about the world around them. It is concerned about uncovering and describing lived experiences and answers the question “What is this like to experience x?” [15,16,17]. Because phenomenology is a person-centered research approach, it was fitting for this study. The main research question involved a phenomenon, the use of smartphone apps for diabetes management and how people perceive their use. To determine if smartphone apps can be an appropriate resource for these individuals, a qualitative study would help to gauge what value this population would assign to such apps and how they would use them.

This study, more specifically, employed interpretative phenomenological analysis (IPA) as its methodology. Experiences, like the use of smartphone apps for diabetes management, cannot be separated from context. IPA assumes that people are always interpreting the world around them through a lens built upon previous experiences, personal beliefs, and current environment [18]. This means that no two people who go through the same event will remember or value the same things. Accordingly, IPA tries to understand how each person makes sense of an experience and how individuals assign different meanings to events in their daily lives [18,19]. IPA is especially suitable for this research question, which sought to understand how a low-income, minority population with diabetes who live in racially segregated communities that are significantly distanced from primary care providers and healthcare facilities (presentation of a certain context) assign value (personal interpretation) to diabetes apps (shared experience).

### 2.3. Interview Process

Data collection for this study came from personal interviews. Since no two people experience diabetes in the same way, patients make individualized decisions regarding their self-care that are tailored to their own different needs. One-on-one interviews are an effective way to collect such personal data [20]. This study employed semi-structured interviews. The interviewer started out with 15 predetermined questions that were asked to all study participants. Table 1 shows the overarching topics these questions addressed. However, the interactions also allowed the interviewer to stray from the set list with spontaneous follow-up questions to allow for further exploration of topics that emerged during the exchange [21].

The University of Tennessee Health Science Center institutional review board provided ethics approval for the study and the entire interview process (protocol code 16-04701-XP). All responses collected were de-identified to maintain confidentiality. Before beginning each interview, the researcher obtained informed consent from every participant. All participants were allowed refusal to answer any interview question they did not wish to address. They were also able to withdraw their consent to participate at any point during the study without any consequences.

### 2.4. Research Site

Interview participants were selected from Methodist South, a 156-bed hospital located in a South Memphis community called Whitehaven [22]. This study sought people with diabetes who had limited access to a primary care provider (PCP) but easy access to a hospital emergency department (ED). Healthcare in the United States is not nationalized. Individuals must have some form of insurance to cover costs or pay out of their own pocket. Additionally, primary care clinics are allowed to refuse care to individuals who cannot afford their services. There are, however, federal laws that ensure hospitals cannot turn away individuals who come to the ED [23]. This means that people who lack access to a regular PCP can and will show up to a hospital ED because they know that the hospital cannot refuse to see them. Methodist South covers a local demographic with such underserved characteristics. This urban community has a predominant minority residency of African Americans (92%) and Hispanics (3%) [24]. The average Whitehaven household income is $46,964, which is approximately $21,000 less than the average household income for the greater-Memphis area ($68,182) [24]. Most notably, this hospital serves a local population with a 45% higher rate of diabetes-related mortality than the city as a whole [25].

### 2.5. Recruitment and Participant Selection

#### 2.5.1. Inclusion and Exclusion Criteria

Inclusion criteria:

- Age ≥ 18;
- Diagnosis of type 1 or 2 diabetes;
- Access to a smartphone that can download and open apps (self-reported);
- Irregular or no contact with a primary care provider (self-reported);
- Recent emergency department visit for a diabetes-related reason.

Exclusion criteria:

- Diagnosis of chemical or drug-induced diabetes or gestational diabetes;
- Inpatient hospitalized for a diabetes-related event without an ED admission;
- Participation in other research studies on diabetes management or treatment.

#### 2.5.2. Selection Process

An ED pharmacist and transitions of care pharmacist at Methodist South worked together to select potential interview participants. First, the ED pharmacist reviewed incoming patients for reason for admission. Then this pharmacist reviewed the patient chart to confirm age and a documented type 1 or 2 diabetes diagnosis. If the information gathered from the chart met the inclusion criteria, the ED pharmacist passed along the patient information to the transitions of care pharmacist. Next, this second pharmacist spoke with the patients to ascertain their PCP status and smartphone access. All patients who met the inclusion criteria received a brief overview of the study. If patients showed interest in participating, they were contacted by the study investigator to arrange a meeting time. Each participant provided one personal interview.

Interview recruitment happened between May 2017 and February 2018. Both the ED pharmacist and transitions of care pharmacist identified a total of 34 patients who met the inclusion criteria. From this patient pool, nine individuals either did not answer when the investigator called or never returned voicemails left requesting to schedule an interview. The investigator was able to schedule an interview with 23 individuals. Of this group, three individuals were a no-show and were not able to reschedule for a later date, and five individuals were ultimately unable to arrange transportation to return to the hospital for an interview. The investigator successfully met and completed interviews with 15 patients. The majority of them, 12 interviewees, had type 2 diabetes, while the remaining three had type 1 diabetes. All interviewees were willing and able to answer the 15 predetermined questions and any follow-up questions that emerged during their individual conversations. 

With personal interviews, the literature does not offer an exact number of participants to recruit. Several qualitative research guidebooks recommend a sample size of five to 15 interviews for phenomenological studies [26,27,28]. More importantly, the study investigator should interview until data saturation occurs. This means that the research should begin to develop themes from the first few interviews collected. If the data from the next interviews fall in line with those existing themes, and no new themes emerge from the later interviews, then the study has reached data saturation [29]. For this study, 15 interviews were sufficient to reach this saturation point.

### 2.6. Data Analysis

Consistent with IPA methodology, this study did not test a predetermined hypothesis. Instead, data analysis involved coding, or the grouping of similar thoughts, ideas, or feelings from the gathered interviews together into clusters. IPA studies like this one involve building themes and understandings from deep analysis of lengthy individual transcripts [26]. Analysis in IPA is called double hermeneutic, or double interpretative [19]. First, the participants described how they would experience a phenomenon, the use of diabetes apps in their daily lives. Then the investigator would try to understand how the participants interpreted and assigned meaning to the experience.

The first step in the analysis process involved familiarization with the data [20,30,31]. This meant repeated listening to the audio recordings of each interview, immediate transcription of each recording, and several readings of the transcribed interviews. After the close readings came the coding process. For this, the researcher began by blocking sections of transcribed text into certain categories. Similar categories were then grouped together based on recurring patterns that emerged. From these clusters of similar or related categories, the researcher became able to identify broader themes [20,31]. These overlying themes were likely to indicate what matters most to patients for diabetes self-management and what would drive them to seek and use mHealth apps.

Key to producing reliable qualitative findings is the acknowledgement of how the researchers’ own biases and motives might influence the study. Termed reflexivity, this is a process by which the researchers perform a self-assessment of their own preconceptions and how this might shape the research process [32]. For this study, the researchers accomplished this by keeping personal journal notes of how their thoughts and beliefs impacted methodological decisions, descriptions of the interview setting, and their subjective thoughts on their relationship with the interviewees [33]. An example of methodological decision-making noted with reflexive journaling was the researchers recognizing a preconception that this underserved population might be sensitive about their lower income, especially when interacting with an interviewer who was working in higher education. Therefore, the researchers decided not to structure any interview questions with exact income figures or ranges in the hopes that interviewees would feel more comfortable and would then open up more about their home experiences with diabetes.

## 3. Results

Data from the interviews produced seven unique thought clusters related to smartphone and app use for diabetes management. As shown in Table 2, these clusters were assembled to generate the following three overall themes:

(1) Despite having little previous knowledge about health apps, patients were all willing to try at least one diabetes-related app;
(2) App functions should be individualized to each patient’s needs for maximum benefit;
(3) Barriers to app use were varied but commonly included knowledge and technological challenges and security issues.

All three prevailing themes are explained further below. For each theme, information from its supporting clusters is presented. Direct interview quotes, edited for grammatical clarity and participant anonymity, are also given to emphasize individual thoughts and viewpoints.

### 3.1. Theme 1: Despite Having Little Previous Knowledge about Health Apps, Patients Were All Willing to Try at Least One Diabetes-Related App

Smartphone usage varied widely among the participants from no or past ownership to current ownership. Of the 15 patients interviewed, three indicated no present ownership. Almost all current smartphone owners used their phones multiple times a day as a part of their daily routines and habits, one interviewee noting that it was “second to nature, havin’ a phone” [personal communication by Participant B, 2017]. Several patients also expressed comfort with their phones beyond making calls and used their phones routinely for other functions like texting, listening to music, accessing social media, and searching the internet.

While the majority of patients interviewed either knew nothing about smartphone mHealth apps or had never used one previously, a few did indicate previous use of a health app. These apps were mostly related to filling or pricing medications. These patients noted a positive experience with prior health app use. One participant, who had a higher comfort level with smartphone use, described a previous experience with a blood glucose app as such:

“I used to have this app where you write down your blood sugar, but I, some kinda way I deleted it. I don’t know why. Um, I’m comfortable in usin’ ‘em. It’s, it’s actually helpful because it usually remind me: did you take your sugar? Or I usually put in my text remind me to check my sugar. And I use the bing bing gotta take your sugar.”

[personal communication by Participant K, 2017]

Other interviewees spoke of the importance of using their smartphone to access the internet to research information about diabetes. Some stated that they turned to the phones whenever they had questions about their chronic condition. One patient described a need to “googled a lot […] whenever I had questions about anything. You know at night, I just googled all the time” [personal communication by Participant J, 2017]. Moreover, another patient described using mobile sites like Google and YouTube to see “what’s gonna happen to somebody, uh, when you not take care of diabetes” and to see “picture of like I don’t wanna end up like that, you know” [personal communication by Participant K, 2017].

All patients, despite their varying comfort levels with using a smartphone, expressed a willingness to try a diabetes health app. Every patient interviewed voiced an understanding that successful diabetes management is not a passive process but one that required their active participation. They understood that using a smartphone health app is a form of active health engagement. There was, therefore, a positive response towards app use from all the interviewees. Additionally, interviewees who did not express frequent or daily use with the smartphone platform also indicated their willingness to invest more time “to put a foot forward,” in becoming acclimated to their mobile devices, especially if “expectin’ a good turnout” [personal communication by Participant B, 2017]. Even though most of these patients knew very little about diabetes apps, there “ain’t nothin’ wrong with tryin’ somethin’ different once” so that if they do “get it workin’ right, ” they “might like it” [personal communication by Participant D, 2017]. Almost all participants said that they would try to use at least one app daily. Additionally, all but one indicated a willingness to pay to download an app if they thought it might be beneficial to their diabetes management.

### 3.2. Theme 2: App Functions Should Be Individualized to Each Patient’s Needs for Maximum Benefit

Participants expressed different challenges in their day-to-day diabetes management. No two interviewees spoke of having the same at-home health routine. While several patients spoke of taking the same brand of insulin, their dosages varied widely, and the times of day they took their medications also differed. One patient might have only need to check his blood glucose once a day, but another required over four daily checks for sufficient glucose monitoring. Even when patients spoke about struggles with a similar topic, like proper diet, their struggles with that issue were different. For example, one patient expressed the following diet-related hardship:

“Well, uh, as far as I can see, it’s like I have to balance. At first, I didn’t worry about what I bought or what I ate, but now, I have to, uh, you know, buy the rights things to, uh, eat when I used to just eat whatever I wanted. But I can’t do that no more. Being on fixed income, uh, I have to just really not, you know, just spend on, on something that I normally would. I have to just spend my money on something that’s more healthy, or, you know, it’s, it’s been a challenge especially since you used to eatin’, you know, whatever you want to. But now you’ve got to be limited, you know, to what you eat, and it’s been a struggle.”

[personal communication by Participant F, 2017]

Another patient, although mentioning the same topic of diet and food, voiced a different concern:

“Oh man, stayin’ away from sweets. You know, like the holidays, like Thanksgivin’, they make ‘em peach cobblers and sweet potato pies. It’s hard, and then when you sittin’ there with all that food […] You know hey I can only have a certain amount of this food. You know what I’m sayin’? And but you wanna try everythang. If you try everything, you gotta get real, real small portions, but then what happen when you get something you really like? Then you eat too much. You know what I’m sayin’? You overindulge. And then you got the sweet stuff. You like, “I’m a try a piece of this” or then, then your sugar’s outta whack.”

[personal communication by Participant O, 2018]

Even though the patients above relayed food-related challenges, their struggles were different. While the first patient might appreciate a diabetes-friendly grocery list/budget tracking app, the second patient might obtain more benefit from a calorie/carb counting app. Therefore, app suggestions and recommendations should include functions that meet that patient’s greatest need(s). One patient may have more use for a diabetes-friendly recipes app to address a challenge of cooking healthier food [personal communication by Participant N, 2018], but another patient might benefit more from a carbohydrate tracking app to help with struggles over portion control [personal communication by Participant I, 2017].

Outside of diet, the same theme of needing app personalization emerged with other facets of diabetes management like medication adherence. Whereas one patient might find great value in an app that reminds him to, “do the insulin” on time [personal communication by Participant J, 2017], another proclaims that he “can keep up with my, you know, what I need to take and when I need to take it. You know, I can pretty much do that myself” [personal communication by Participant A, 2017]. Based on these interviewee responses, no one overarching app can be used to help all patients with their at-home diabetes management. App selection must be tailored to each patient’s specific challenges and needs. For some patients, this could mean just choosing one app that addresses the part of self-management that they struggle with most, e.g., medication reminder. For others, suggesting apps that include multiple functions like a combination glucose and diet tracker, may be more helpful. Mostly importantly, all apps recommended should include functions that address the facet of diabetes self-management with which the individual needs the most help.

### 3.3. Theme 3: Barriers to App Use Were Varied but Commonly Included Knowledge and Technological Challenges and Security Issues

Many patients stressed the importance of an app as being user-friendly, something “not hard” or “super complicated” [personal communication by Participant B, 2017]. This meant that they did not want something that required extensive training or included advanced medical jargon. Those less comfortable with operating a smartphone expressed hesitance at being able to use an app effectively. These patients would need for someone, either a hospital provider or a family member, to guide them through using an app.

Other concerns noted were related to privacy and security. Interviewees expressed concerns about inputting personal data in an app and theft of that information. Most felt comfortable in giving up health metrics like weight or blood glucose reading but were leery about disclosing any financial information. Those who currently owned a smartphone were more likely to bring up technological challenges like storage space or frequent need for updates. They expressed a desire for an app that would run smoothly “on the regular” without crashing their phones or have “somethin’ happen […] where it quit workin’” [personal communication by Participant N, 2018]. A couple of patients also brought up fears of too much spam or viruses.

## 4. Discussion

Personal interviews from low-income, minority patients with diabetes indicated an interest in using smartphone mHealth apps. These patients had widely different baseline exposure levels with smartphones, ranging from only seeing those in their immediate household use the phones to consistent, daily use. Nonetheless, all interviewees stated they were open to using a diabetes-related app. Even those with very little or no previous exposure to a smartphone voiced a willingness to try at least one app at home; their interest in seeing if mHealth could benefit their diabetes management overrode any hesitance over using unfamiliar technology.

Patients mentioned common hurdles related to medication affordability and adherence, proper diet and exercise, and fluctuating blood glucose management. Thus, they demonstrated interest in app features related to diet, exercise tracking, and medication management. This is similar to findings from previous survey questionnaire studies where patients indicated app preferences for activity tracking, medication reminders/alarms, carbohydrate counting, and blood glucose tracking [34,35]. Participant struggles, however, were individualized to their diabetes progression and their current living situation. For instance, several patients noted struggles with their diets, but they might not benefit from using the same diet-related app. To provide the greatest benefit to this underserved population, app selection should be tailored to address each patient’s specific at-home need.

Participants also expressed varying concerns over app use. These ranged from user-oriented fears, i.e., inadequate technological knowledge to work an app, to app-oriented concerns, i.e., data security or virus attacks. In line with a previous survey study [34], the majority of interviewees said that they would be willing to pay for an app and so cost to download would not be a barrier to use.

Limitations of this study may include an inability to generalize results beyond the Memphis, TN, USA, population, as the context for these patient experiences may be different compared to other locations and settings. Because the majority of patients interviewed had type 2 diabetes, generalizability may also be limited for type 1 diabetes. Additionally, findings from this study do not extend to non-commercially available apps, such as those attached to a hospital electronic medical record or a closed health system. Because this was a small, exploratory study, no correlations could be drawn between app interest and specific patient characteristics like disease severity or duration.

Selection bias may have also affected results from this study. First, the hospital charts did not routinely collect PCP information. Patients, therefore, self-reported whether or not they regularly visited a PCP for their diabetes. The study was then only able to collect interviews from those who were truthful about this and was not able to gather information from patients who truly had no PCP but told the screening pharmacist that they did. The patients who agreed to participate and were able to come to an interview might have also been more inherently motivated to manage their diabetes and to express a willingness to try an app. The interviewer worked to established trust with the participants by clearly outlining before each interaction that there were no right or wrong answers and that all answers were to remain confidential and anonymous. Nonetheless, participants could have still felt compelled to please the interviewer by expressing interest in using an app, even if they truly had no desire to try one. The inclusion criteria of a diabetes-related ED visit could have also influenced the participants’ responses. With the shock of a health event fresh in mind, interviewees may have felt more compelled to express a willingness to use smartphone apps than if they had not experienced a recent diabetes-related ED admission.

## 5. Conclusions

An intensive search of the literature revealed no previous qualitative studies about leveraging smartphone apps to address diabetes-related health disparities. Results from this study show a potential for mHealth apps to be a valuable resource in an underserved patient population. Therefore, the findings from this study indicate a need for further research in engaging vulnerable patient populations with mHealth. This could include future pilot studies examining clinical effectiveness on blood glucose levels when making a personalized app selection for patients to use at ED discharge. Additional studies could examine the cost effectiveness of having underserved patients use smartphone apps at home to prevent diabetes-related ED readmissions.

## Figures and Tables

**Table 1:** Summary of topics covered by the predetermined interview questions.

Topic	Number of Questions
diabetes health status and self-management behaviors	4
current smartphone usage	3
interest in smartphone apps	6
obstacles to app use	2

**Table 2:** Summary of overall mHealth themes derived from all personal interviews with their supporting clusters.

Overall Theme	Clusters	Subclusters
Despite having little previous knowledge about health apps, patients were all willing to try at least one diabetes-related app.	Openness to health apps	Number of apps Payment
	Comfort using smartphones	
	Current or previous phone usage	
	Knowledge about apps	
App functions should be individualized to each patient’s needs for maximum benefit.	App functions interest	
	Challenges to management	
Barriers to app use were varied but commonly included knowledge and technological challenges and security issues.	Concerns about app use	
	Comfort using smartphones	

## Data Availability

Data presented in this study may be available upon request from the corresponding author.
